# Protective Effect of JXT Ethanol Extract on Radiation-Induced Hematopoietic Alteration and Oxidative Stress in the Liver

**DOI:** 10.1155/2018/9017835

**Published:** 2018-10-28

**Authors:** Xian-Zhe Dong, Yu-Ning Wang, Xiao Tan, Ping Liu, Dai-Hong Guo, Can Yan

**Affiliations:** ^1^Center of Drug Security, General Hospital of Chinese PLA, Beijing 100853, China; ^2^Department of Surgery, Chinese PLA General Hospital, Beijing 100853, China; ^3^Department of Basic Theory of Chinese Medicine, School of Pre-Clinical Medicine, Guangzhou University of Chinese Medicine, Guangzhou 510060, China; ^4^The Research Centre of Basic Integrative Medicine, Guangzhou University of Chinese Medicine, Guangzhou 510060, China

## Abstract

This study aims at investigating the radioprotective effect of ethanol extract from Ji-Xue-Teng (JXT, *Spatholobus suberectus*) on radiation-induced hematopoietic alteration and oxidative stress in the liver. Mice were exposed to a single acute *γ*-radiation for the whole body at the dose of 6.0 Gy, then subjected to administration of amifostine (45 mg/kg) or JXT (40 g crude drug/kg) once a day for 28 consecutive days, respectively. Bone marrow cells and hemogram including white cells, red cells, platelet counts, and hemoglobin level were examined. The protein expression levels of pJAK2/JAK2, pSTAT5a/STAT5a, pSTAT5b/STAT5b, and Bcl-2 in bone marrow tissue; levels of reactive oxygen species (ROS); and the activity of superoxide dismutase (SOD), malondialdehyde (MDA), and glutathione peroxidase (GSH-Px) in serum and liver tissue were determined. At the end of the experiment, the effect of JXT on cell viability and G-CSF and G-CSFR levels in NFS-60 cells were tested by CCK-8 assay, ELISA, and flow cytometry. The results showed that the mice exposed to *γ*-radiation alone exhibited a typical hematopoietic syndrome. In contrast, at the end of the 28-day experiment, irradiated mice subjected to oral administration of JXT showed an obvious improvement on blood profile with reduced leucopenia, thrombocytopenia (platelet counts), RBC, and hemoglobin levels, as well as bone marrow cells. The expression of pJAK2/JAK2, pSTAT5a/STAT5a, and Bcl-2 in bone marrow tissue was increased after JXT treatment. The elevation of ROS was due to radiation-induced toxicity, but JXT significantly reduced the ROS level in serum and liver tissue, elevated endogenous SOD and GSH-PX levels, and reduced the MDA level in the liver. JXT could also increase cell viability and G-CSFR level in NFS-60 cells, which was similar to exogenous G-CSF. Our findings suggested that oral administration of JXT effectively facilitated the recovery of hematopoietic bone marrow damage and oxidative stress of the mice induced by *γ*-radiation.

## 1. Introduction

Radiotherapy is widely used in cancer treatment. Although irradiation is targeted at malignant tissues, the surrounding normal tissues or organs are also affected [1–3]. Whole-body radiation may result in acute radiation syndrome with profound pathophysiological consequences characterized as severe suppressed hematopoiesis, gastrointestinal injury, cerebrovascular system, and neurological damage [4–7]. The hematopoietic system is exceedingly sensitive to ionizing radiation, with an associated decrease in bone marrow cells (BMC) and circulating peripheral blood cells that may further cause anemia, bleeding, infection, and declined immune function [8, 9]. According to previous reports, oxidative damage to DNA can be a result from radiation-induced free radicals or ROS [10]. The ionizing radiation at a relatively high level can lead to changed gene expression pattern and mutation prevalence and blunt the repairing mechanisms of DNA damage [11, 12]. The development of efficacious radioprotectors to accelerate hematopoiesis recovery should be a valuable contribution to radiation oncology.

As a consequence, it is essential to search radioprotectors to control side effects in both planned exposure such as radiotherapy and unplanned exposure such as natural radiation [13]. Although synthetic compounds including thiols, baminothiols, thiadiazoles, or benzothiazoles reveal certain radioprotective effects, their side effects have also gained extensive attention [14]. Amifostine is the only agent approved so far by the Food and Drug Administration for treating radiation syndrome [15]. However, amifostine gives rise to significant adverse reactions in many cases; for example, a phase III clinical trial has found its adverse reactions in 41% of head and neck cancer patients, with symptoms including hypotension, hypocalcemia, nausea, vomiting, and allergy [16]. Therefore, it is a promising strategy to develop natural radioprotectors from plants and herbs [17, 18]. Herb-derived phytochemicals offer opportunities to develop efficacious radioprotective agents due to low toxicity and few side effects [19–21]. Recently, natural compounds such as genistein or ginsan from plant extracts have been extensively explored to have strong capacity to alleviate radiation-induced damage [2, 22].

Ji-Xue-Teng (JXT) is a traditional herb from the dry rattan of *Spatholobus suberectus* (*S. suberectus*) Dunn, recorded in Gang Mu Shi Yi as a holy drug of the blood, which can remove blood clot and produce fresh blood. Modern pharmacological researches have documented that JXT and its active components are widely used for improving blood circulation and executing anti-inflammation, antibacterial, neuroprotective, antioxidative, and anticancer effects [23–25]. According to our previous report, the bioactive components of JXT can promote the proliferation of hematopoietic progenitor cells (HPC) in bone marrow-depressed mice [26], but the impact and underlying molecule mechanism of JXT on irradiation-induced hematological toxicity and other organ injury are still unclear. In the present study, the effect of JXT on 6.0 Gy *γ*-radiation-induced myelosuppression and oxidative damage of the liver tissues in mice and the underlying hematopoietic mechanisms were explored.

## 2. Materials and Methods

### 2.1. Chemicals

JXT was purchased from Beijing Lvye Pharmaceutical Co. Ltd. (Beijing, China), Fujian (China) origin. JXT was cut into small pieces and soaked in 75% ethanol at 8 times volume overnight and then extracted 3 times with 1 h for each time. Finally, the filtrates were mixed and concentrated by a rotary evaporator with bath temperature lower than 40°C. Ethanol extract was lyophilized to powder.

The reagents and kits for determining enzyme activity were purchased from Nanjing Jiancheng Bioengineering Institute (NJBI, Nanjing, China). The antibodies for evaluating G-CSFR, Bcl-2, JAK2, pJAK2, STAT5a, pSTAT5a, STAT5b and pSTAT5b were purchased from Abcam (Abcam, USA). Amifostine was purchased from Dalian Merro Pharmaceutical Factory (Dalian, China); RPMI-1640 culture medium and fetal bovine serum (FBS) were purchased from HyClone (HyClone, USA). G-CSF ELISA kit was purchased from Shanghai Tongwei Biotech Co. Ltd. (Shanghai, China).

### 2.2. Animals

A total of 40 Chinese Kun Ming (KM) mice with the age of 6–8 weeks and inbred colony were purchased from the Laboratory Animal Breeding and Research Center in PLA General Hospital, Beijing, China, and maintained under controlled conditions of temperature (23 ± 2°C) and light (12L : 12D). All mice were provided with standard chow diet and water ad libitum.

### 2.3. Irradiation and Administrations

Gamma radiation (*γ*-radiation) at the dose of 6 Gy was delivered in the Department of Radiotherapy and Oncology, the Military Medical Academy of Science. The mice were randomly divided into 4 groups including nonirradiation control, irradiation control, positive treatment control, and JXT treatment groups with 10 mice in each group. The mice from the positive treatment control group were administrated with amifostine at 43.6 mg/kg by tail vein injection [27], and the mice from the JXT treatment group were orally administrated with JXT ethanol extract at the dose of 40 g/kg based on the amount of crude drug. JXT was dissolved in sterile saline and orally administrated for 21 consecutive days after mouse irradiation.

### 2.4. Hematological Analysis

The effect of JXT extract on the peripheral blood of mice from each experiment group was analyzed. On day 21 after irradiation, hemoglobin (HGB) level, erythrocyte (RBC) count, total leukocyte (WBC) count, and platelet (PLT) count for each mouse were measured at 0 day (before irradiation) and 21 days postirradiation using an automated hematology analyzer (BC-2800vet; Mindray, China).

### 2.5. Antioxidant Activity

The ROS level in the serum and ROS, SOD, GSH-Px, and MDA levels in the liver tissue were analyzed, respectively, according to the instructions of the kits from the manufacturer.

### 2.6. Bone Marrow Cell Counting

Bone marrow cells were collected from both femurs of each mouse at day 10 after whole-body radiation at the dose of 6 Gy. The collected bone marrow cells were rinsed with PBS, and single-cell suspension was obtained. After the lysis of red blood cells, the bone marrow cells were suspended in RPMI-1640 with 10% fetal bovine serum (FBS) and 100 U/ml antibiotics. Then, the bone marrow cells were counted in a hemacytometer under an optical microscope to determine whether JXT extract can protect the bone marrow cells from the depletion induced by *γ*-radiation.

### 2.7. Western Blot

The preparation of bone marrow cells was the same as above. For western blot analysis, total protein was extracted first, and cells were washed twice with cold PBS and lysed in the radioimmunoprecipitation assay (RIPA) buffer (20 mM Tris-HCl, pH 7.5; 150 mM sodium chloride; 1% Triton; 1 mM EGTA; 1 mM EDTA; 2.5 mM sodium pyrophosphate; 1 mM *β*-glycerophosphate; 1 mM sodium orthovanadate; 1 *μ*g/ml leupeptin; and 1 mM PMSF) for 20 min on the ice. After centrifugation at 12000×*g* for 10 min, protein was then quantitated by using a bicinchoninic acid protein assay kit (Bioworld Technology, USA). After being heated at 95°C for 8 min, the samples were loaded on 12% SDS-polyacrylamide gel for electrophoresis and transferred to PVDF membrane (Millipore, Billerica, MA). The membrane was blocked for 2.5 h in TBS-T buffer (10 mM Tris, pH 8.0, 150 mM NaCl, and 0.1% Tween-20) containing 3% (*w*/*v*) bovine serum albumin (BSA; Sigma-Aldrich, USA) at room temperature and then probed with each primary antibody (1 : 500 dilution) at 4°C overnight. After washing with TBS-T for three times, the membrane was then incubated with appropriate horseradish peroxidase-labeled secondary antibody for 2 h at room temperature. For quantitative analysis of immunoblot bands, the densities of the bands were measured by scanning densitometry (Bio-Rad, Hercules, CA). All experiments were repeated at least three times.

### 2.8. Cell Culture

NFS-60 cell line, derived from murine myeloid leukemia, is responsive to different growth factors including G-CSF. Cells were maintained in RPMI 1640 medium supplemented with 10% FBS, 50 U/ml penicillin, 50 *μ*g/ml streptomycin, and 15 ng/ml G-CFS (Sigma, USA).

### 2.9. Cell Viability Detection by CCK-8 Assay

NFS-60 cell viability after JXT treatment was evaluated with CCK-8 assay. Cells (3000 cells/well) were plated in 96-well plates. After 24 h culture, JXT at different final concentrations (1.6, 8, 40, and 200 g/ml) was added into the plates and detected after 24 h culture. Briefly, 20 *μ*l of CCK-8 solution was added to the culture medium and incubated at 37°C for 4 h. Then, the OD was spectrophotometrically measured at 450 nm using a microplate spectrophotometer (1420 Victor^3^, Perkin-Elmer, USA). Viability was expressed as the percentage of vehicle-treated (basal) culture that was set to 100%.

### 2.10. Enzyme-Linked Immunosorbent Assay

Culture media were collected following treatments and promptly stored at −80°C until the future assay. The G-CSF level was measured using commercially available paired antibody quantitative ELISA kit according to the manufacturer's instructions. Data were expressed as 10^−6^ *μ*g/ml. Dispensed antigen standards and samples were added to each well of 96-well plates precoated with primary antibodies. After adding biotin conjugate reagent and enzyme conjugate reagent into each well, the plates were incubated at 37°C for 30 min. Then, the plates were rinsed with distilled water for 5 times and measured using a microtiter plate reader (Perkin-Elmer, USA).

### 2.11. Flow Cytometric Detection of G-CSFR

The G-CSFR level in cells was measured using flow cytometry. A total of 5 × 10^6^ NFS-60 cells were plated in 6-well plates for each well. After treatment with JXT at different final concentrations (1.6, 8, 40, and 200 *μ*g/ml) for 24 h, the cells were harvested and washed in PBS and then centrifuged at 1000×*g* for 5 min. The cell pellet was resuspended at the density of 5 × 10^6^ cells/ml and incubated with G-CSFR antibody for 30 min at 37°C. Then, the cells were washed in PBS and incubated with IgG secondary antibody for 30 min at 37°C. The cells were then analyzed in a Becton Dickinson flow cytometer (Becton, Dickinson and Company, USA). Each sample was collected at the amount of ten thousand cells.

## 3. Statistical Analysis

All data were expressed as mean ± standard deviation (M ± SD). Statistical analysis was conducted by using SPSS 16.0 software. One-way analysis of variance (ANOVA) followed by Tukey's post hoc test was used for multiple group comparisons. The statistically significant difference was considered at *P* < 0.05.

## 4. Results

### 4.1. Effect of JXT Treatment on the Loss of Body Weights and Organ Indexes

**Figure 1:**
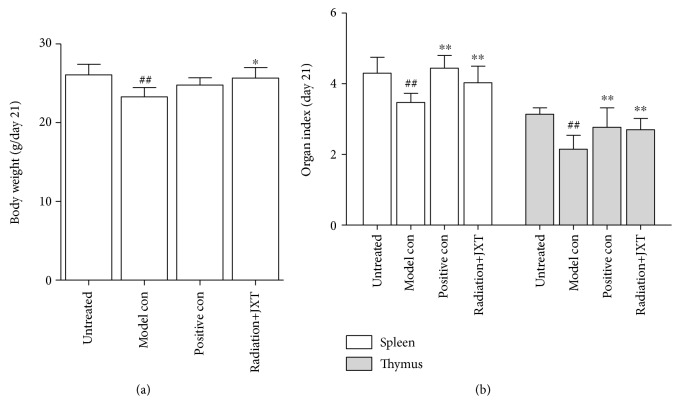
Effect of JXT on body weights of Co^60^-irradiated mice. All data were expressed as mean ± SEM (*n* = 10). ^#^*P* < 0.05; ^##^*P* < 0.01 when compared with the untreated control group. ^∗^*P* < 0.05; ^∗∗^*P* < 0.01 when compared with the Co^60^-irradiated group. (a) Body weight; (b) organ index.

Figure 1 demonstrated the changes of body weights, spleen index, and thymus index in each group at day 21. There was a profound loss in body weight, spleen index, and thymus index of the animals exposed to 6.0 Gy *γ*-radiation. The administration with JXT significantly increased the body weight (*P* < 0.05), spleen index, and thymus index (*P* < 0.01) when compared to the radiation group. The results suggested that JXT administration played a positive role in preventing radiation-induced loss of body weight, spleen index, and thymus index of the mice.

### 4.2. Effect of JXT Treatment on Peripheral Blood Cells

**Figure 2:**
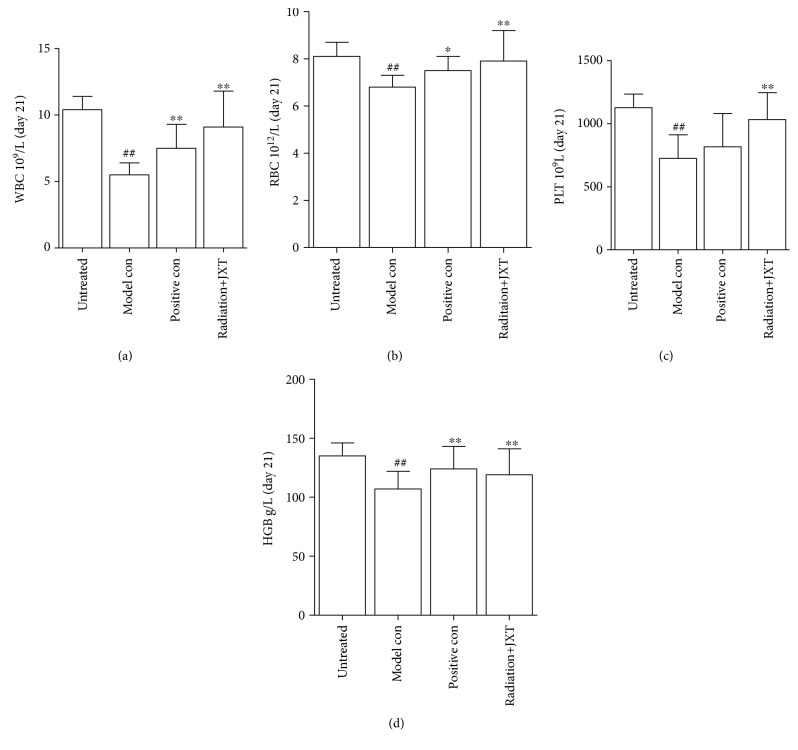
Effect of JXT on the number of circulating peripheral blood cells in Co^60^-irradiated mice. All data were expressed as mean ± SEM (*n* = 10). ^#^*P* < 0.05; ^##^*P* < 0.01 when compared with the untreated control group. ^∗^*P* < 0.05; ^∗∗^*P* < 0.01 when compared with the Co^60^-irradiated group. (a) White blood cell (WBC) number; (b) red blood cell (RBC) number; (c) platelet (PLT) number; (d) hemoglobin (HGB) level.

WBC counts of the mice before and after irradiation were shown in Figure 2(a). Total WBC counts in irradiated animals were determined to be significantly reduced at day 21 (*P* < 0.01) when compared with those in the control group. The administration with JXT and amifostine could attenuate the decrease in WBC counts.  Figure 2(b) showed that irradiation reduced the RBC number at day 21, while a significant increase in the RBC number was observed on day 21 in the JXT-treated mice (*P* < 0.01) and amifostine-treated mice (*P* < 0.05). As shown in Figure 2(c), the number of peripheral PLT decreased markedly after irradiation, and the PLT decline was significantly attenuated by JXT on day 21 in comparison to the radiation group (*P* < 0.01).  Figure 2(d) showed that irradiation reduced the HGB level at day 21; in contrast, a significant increase in the HGB level was observed on day 21 in the JXT- and amifostine-treated mice (*P* < 0.01 and 0.05). These results showed that the mice exposed to *γ*-radiation alone exhibited a typical hematopoietic syndrome. On the other hand, at the end of the 21-day experiment, irradiated mice receiving oral administration of JXT showed an improved blood profile with reduced leucopenia, thrombocytopenia (platelet counts), RBC, and hemoglobin levels.

### 4.3. Effect of JXT Treatment on the Depletion of Bone Marrow Cells

**Figure 3:**
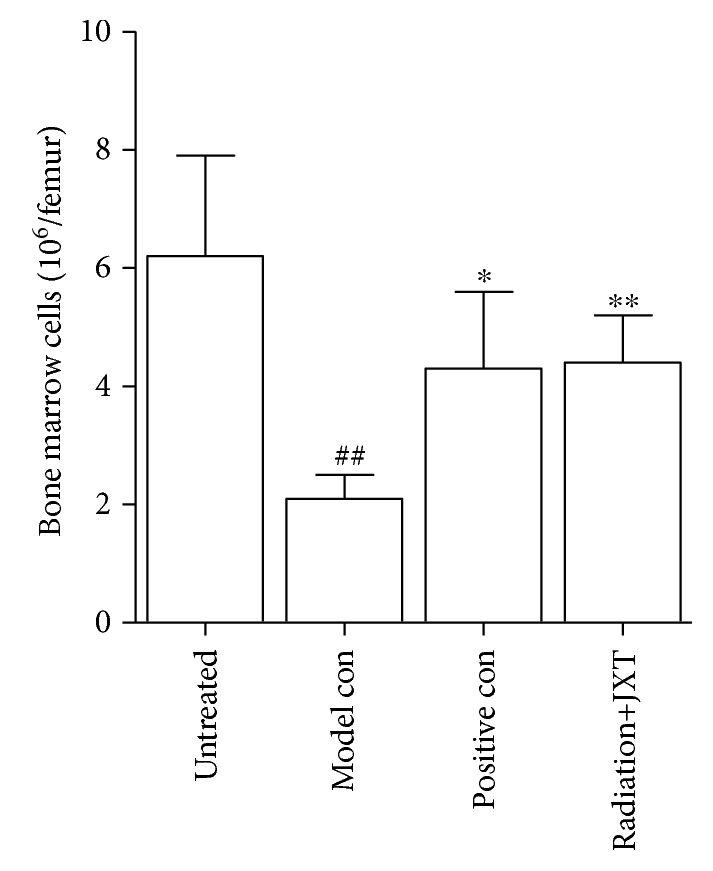
Effect of JXT on the number of bone marrow cells in Co^60^-irradiated mice. All data were expressed as mean ± SEM (*n* = 10). ^##^*P* < 0.01 when compared with the untreated control group. ^∗^*P* < 0.05; ^∗∗^*P* < 0.01 when compared with the Co^60^-irradiated group.

Subsequently, the potential of JXT to protect irradiation-induced damage of bone marrow cells was also investigated. The effect of JXT on hematopoietic tissue in irradiated mice was evaluated by HE staining in our previous study [28]. The marrow cellularity in the radiation group was decreased when compared with that in the naive group. Although the irradiation can result in a continuous increase in the number of adipocytes [29], JXT treatment could significantly increase the total number of bone marrow cells and ameliorate cellular contents of the bone marrow (Figure 3, *P* < 0.01).

### 4.4. Effect of JXT Treatment on the Expression of Proteins in JAK/STAT Signal Pathway

**Figure 4:**
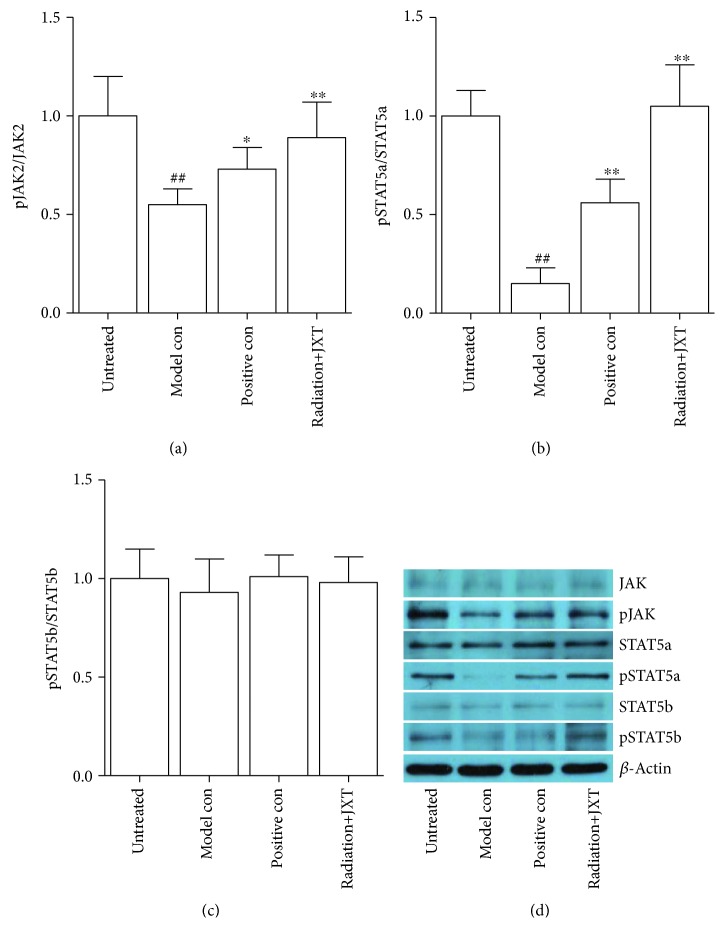
Effect of JXT on the phosphorylation of JAK2, STAT5a, and STAT5b in Co^60^-irradiated mice. All data were expressed as mean ± SEM (*n* = 10). ^##^*P* < 0.01 when compared with the untreated control group. ^∗^*P* < 0.05; ^∗∗^*P* < 0.01 when compared with the Co^60^-irradiated group.

In order to identify whether JAK/STAT signal pathway can be activated by JXT, we examined the phosphorylation of JAK2, STAT5a, and STAT5b in the bone marrow after JXT treatment. The expression of pJAK2/JAK2 and pSTAT5a/STAT5a in bone marrow tissue was decreased after radiation, and the expression of phosphor-JAK2 and phosphor-STAT5a was enhanced. But the expression of pSTAT5b/STAT5b was not changed after radiation and JXT treatment (Figure 4).

### 4.5. Effect of JXT Treatment on the Proliferation, G-CSF, and G-CSFR of NFS-60 Cells

**Figure 5:**
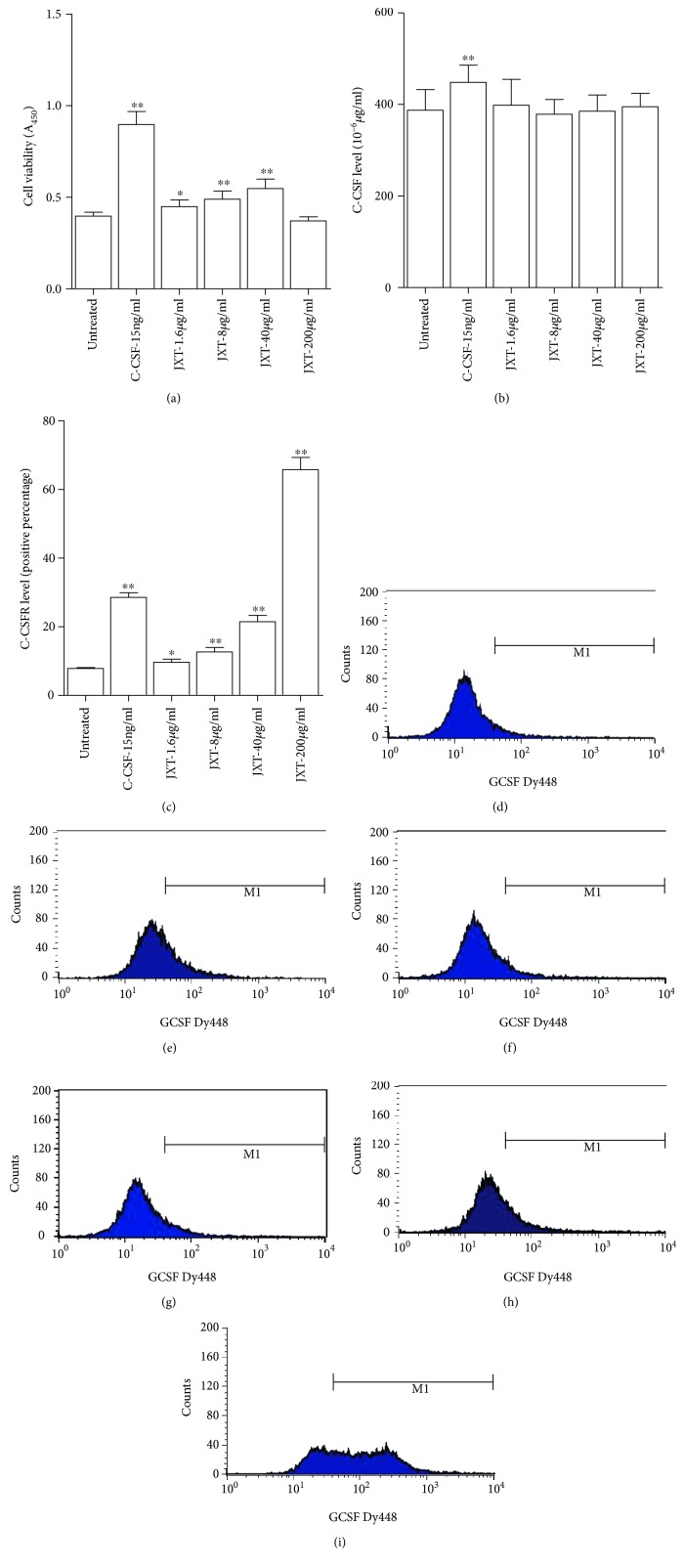
Effect of JXT on cell viability and G-CSF and G-CSFR levels in NSF-60 cells. All data were expressed as mean ± SEM (*n* = 3). ^∗^*P* < 0.05; ^∗∗^*P* < 0.01 when compared with the untreated control group.

The CCK-8 assay was used to evaluate the effect of JXT on the viability of NFS-60 cells. The cells treated with G-CSF (15 ng/ml) revealed an obvious increase in the percentage of viable cells when compared to the control group. After JXT treatment at different concentrations (6 *μ*g/ml to 40 *μ*g/ml), the viability of NFS-60 cells was increased in a concentration-dependent manner (*P* < 0.05 or *P* < 0.01). However, the viability of NFS-60 cells in the 200 *μ*g/ml treatment group was suppressed (Figure 5(a)). At the same time, we measured the G-CSF level in the same conditioned media by ELISA. Only 15 ng/ml G-CSF could induce the release of G-CSF (*P* < 0.01), but no change in the JXT treatment groups (Figure 5(b)). Flow cytometry showed that JXT treatment at 6–200 *μ*g/ml could also increase the G-CSFR level in NFS-60 cells (Figures 5(c)–5(i)).

### 4.6. Effect of JXT Treatment on ROS, SOD, GSH-Px, and MDA in the Serum and Liver

**Figure 6:**
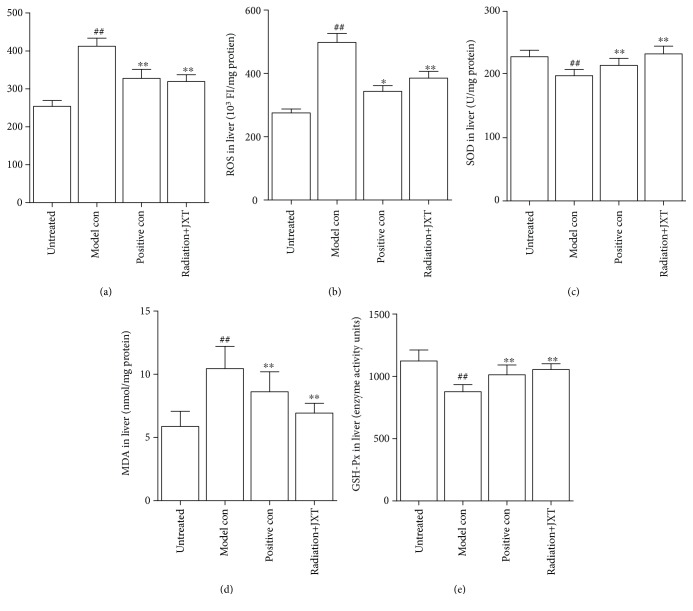
Effect of JXT on the levels of GSH-Px, SOD, and MDA in Co^60^-irradiated mice. All data were expressed as mean ± SEM (*n* = 10). ^##^*P* < 0.01 when compared with the untreated control group. ^∗^*P* < 0.05; ^∗∗^*P* < 0.01 when compared with the Co^60^-irradiated group.

We next investigated whether JXT can regulate oxidative stress system in mice under radiation. JXT treatment significantly reduced the ROS level that was elevated due to radiation-induced toxicity in the liver and serum (Figures 6(a) and 6(b)) and improved endogenous SOD and GSH-Px levels, as well as reduced MDA level in the liver (Figures 6(c)–6(e)).

### 4.7. Effect of JXT Treatment on Bcl-2 Expression in the Bone Marrow

**Figure 7:**
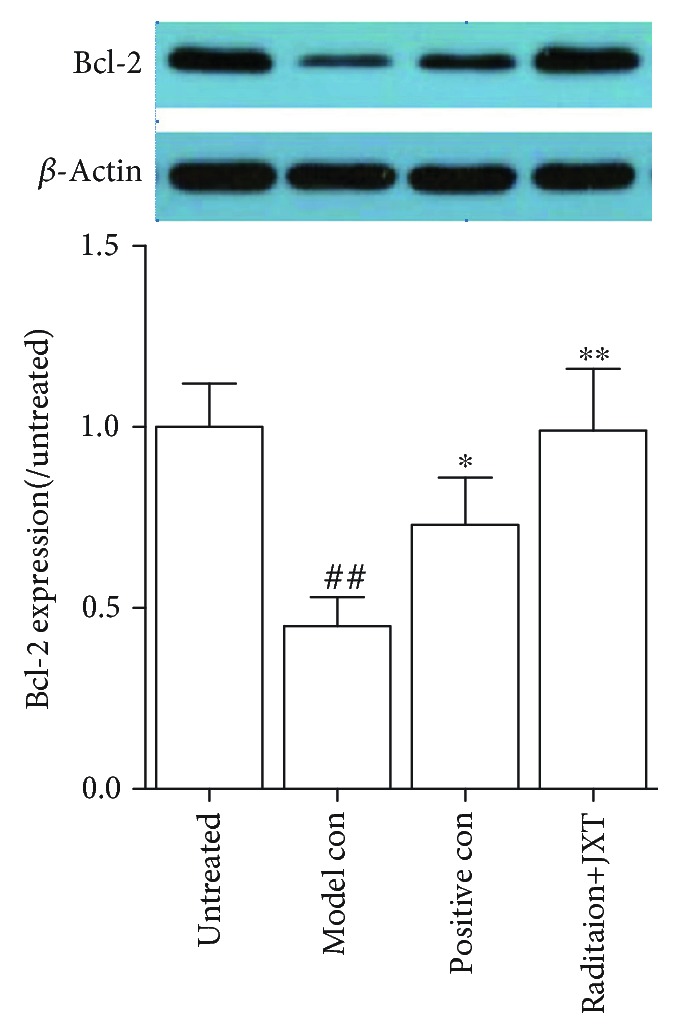
Effect of JXT on the expression of Bcl-2 in Co^60^-irradiated mice. All data were expressed as mean ± SEM (*n* = 10). ^##^*P* < 0.01 when compared with the untreated control group. ^∗^*P* < 0.05; ^∗∗^*P* < 0.01 when compared with the Co^60^-irradiated group.

In order to explore the effect of JXT on the apoptosis of bone marrow cells from mice upon *γ*-radiation, we evaluated the expression levels of Bcl-2 protein before and after JXT treatment. As shown in Figure 7, the expression of Bcl-2 was significantly declined in the radiation-treated mice, and JXT could increase the expression of Bcl-2 in bone marrow tissue significantly (*P* < 0.01).

## 5. Discussion

The bone marrow is the most important hematopoietic organ with hematopoietic cells at different developmental stages. A high proliferation rate of bone marrow cells is highly susceptible to irradiation-induced injury [30]. Bone marrow injury can cause the suppression of hematopoiesis, thus resulting in the low number of circulating blood cells and undesirable side effects such as anemia, bleeding, and infection [31, 32]. We previously demonstrated the protection of bioactive components in JXT against radiation-induced bone marrow damage [26], and herein we have applied an irradiation-induced myelosuppressed mouse model to investigate the protection potential of ethanol extract from JXT on radiation-induced hematological toxicity. Results showed that the administration with JXT could ameliorate Co^60^ irradiation-induced damage and significantly increase the number of peripheral blood cells (WBC, RBC, and PLT), body weight, spleen index, thymus index, and bone marrow cells. Together, these results confirm the enhancement of JXT on hematopoiesis.

Normally, the peripheral blood cell number maintains relative stability, depending on the hematopoietic function of the bone marrow [33]. Radiation exposure can cause injury in hematopoiesis and can result in peripheral blood cell cytopenia and the decrease in mature RBC in anemia. The decreased PLT can lead to bleeding, and the decreased WBC can result in infection [5]. Our results demonstrate that Co^60^ irradiation significantly reduced WBC and PLT counts in the peripheral blood of the mice; however, JXT administration can accelerate the recovery of peripheral blood cells. In order to investigate the effect of JXT treatment on bone marrow injury, the analysis of BMC counts indicated that Co^60^ irradiation significantly decreased the BMC number, and JXT treatment attenuated this reduction, suggesting that JXT may accomplish the protection by improving the density of BMC and increasing the number of nucleated cells in the bone marrow of irradiated mice.

In the present study, we confirmed the radioprotective effect of JXT extract in the hematopoietic system *in vivo* and *in vitro*, and the alcohol extract of JXT can promote protein phosphorylation and the activation of JAK2-STAT5 signal pathway, thereby promoting hematopoiesis. The regulation of hematopoiesis involves multiple signal transduction pathways, in which the JAK-STAT signal pathway is an important signal pathway for the signal transduction of hematopoietic growth factors [34, 35]. Receptor dimerization induced by cytokine and receptor binding can lead to JAK phosphorylation and initiate signal transduction. The activated JAK then catalyzes substrate proteins such as STAT, transmits the information to nuclei, and regulates gene expression, cell proliferation, and differentiation [36–38]. The signal transduction of G-CSF and GM-CSF receptors can be completed through the JAK2-STAT5 signal pathway [39].

In our studies, Bcl-2 was decreased after radiation treatment and increased in JXT-treated mice. The signal transducer and activator of transcription 5 (STAT5) are a critical regulator of normal and leukemic lymphomyeloid development through activating downstream early-acting cytokines, their receptors, and Janus kinases (JAKs) [40–43]. STAT5-mediated regulation of Bcl-2 in hematopoietic cells has been reported both in mouse [44] and in human hematopoietic cells [45]. STAT5ab^ΔN/ΔN^ mast cells have a reduced level of Bcl-2 expression. Bcl-2 is a bona fide direct target of STAT5 so that STAT5 requires the N-domain for the suppression of miR15/16, induction of Bcl-2, and induction of survival signaling in mast cells and myeloproliferative neoplasms (MPNs) [46, 47]. The overexpression of Bcl-2 increases both stem cell number and repopulation potential [48–50]. Bcl-2 also has the antiapoptotic function so that the deficient survival function of BMC can be restored by adding Bcl-2 after JXT treatment.

Ionizing radiation can produce free radicals in cells, such as superanion, hydroxyl, and hydrogen peroxide free radicals [51]. The activity of free radicals can directly or indirectly damage the cell membrane and nucleic acid in the body, thus causing DNA strand breaks and the changes in tissue morphology and metabolism function, enzyme activity, and microcirculation. If free radicals are not timely removed, they could result in more serious physical or chemical injury [52, 53]. Radiation-induced liver injury often occurs in abdominal and pelvic tumors. The radiation-induced injury of the liver results in cell degeneration, apoptosis and necrosis, disordered enzyme activity, biofilm destruction, metabolic disorders, and severe functional failure of liver. Under normal circumstances, free radicals in the human body are in the dynamic balance of continuous production and scavenging. The scavenging of free radicals is mainly dependent on the action of antioxidant enzymes. SOD is a metal enzyme widely existed in organic organisms. It has super antioxidant function and can catalyze disproportionation of free radicals and scavenge free radicals, so as to protect damaged cells and maintain dynamic balance of oxidation and antioxidant reactions. MDA is the degradation product of lipid peroxide, and it has an attack on body cells. The content of MDA can reflect the extent of lipid peroxidation and indirectly reflect the damage of cells. The interaction of MDA and SOD has demonstrated that SOD activity can indirectly reflect the scavenging capacity of oxygen free radicals. MDA content can indirectly reflect free radical-induced damage degree. In the present study, JXT could regulate the oxidative stress system of mice under radiation, suggesting that JXT can protect the liver from oxidative damage induced by radiation.

Many natural products and compounds have been found to have radioprotective effects. A novel lignin-derived polyphenolic composition with ammonium molybdate, BP-C2 mitigates radiation-induced damage in midlethal range of radiation doses. Effects are mediated by the enhancement of extramedullar hematopoiesis in the spleen and a protective effect on the intestinal epithelium [54]. Tea polyphenols, particularly in the combination in TP50 as the radioprotector in mice, have antioxidant potential activity and can reduce inflammatory cytokines especially during the recovery of the hematopoietic system [55]. Beetroot has the potency to preserve bone marrow integrity and stimulate the differentiation of HSCs against ionizing radiation [56].

In our study, JXT has a protective function against hematopoietic dysfunction induced by Co^60^ radiation. JXT treatment can improve bone marrow damage and ameliorate cellular contents of the bone marrow through stimulating the JAK2/STAT5a signal pathway, thus increasing Bcl-2 expression and blocking bone marrow cell apoptosis in Co^60^-irradiated mice. The antioxidant capacity of JXT in the liver can be attributed to the regulatory effect on SOD, GSH-Px, and MDA. Therefore, JXT may be a promising radioprotective agent for the suppression of hematopoiesis.

## Data Availability

The data used to support the findings of this study are available from the corresponding author upon request.
